# Dr. Frank Savary Pearce

**Published:** 1904-08

**Authors:** 


Dr. Frank Savary Pearce, of Philadelphia, died at Stubenville, Ohio, May 27, 1904, aged 36 years. He graduated at the University of Pennsylvania and at the time of his death was professor of neurology in the Medico-Chirurgical College of Philadelphia. He was the author of a textbook on nervous diseases and was preparing another on mental diseases which was soon to issue from the press. His illness was brief and his sudden death was a shock to a wide circle of professional and other friends.

